# NC-OFDM Satellite Communication Based on Compressed Spectrum Sensing

**DOI:** 10.3390/s22103800

**Published:** 2022-05-17

**Authors:** Yong Wang, Hehao Niu, Qingsong Zhao, Lei Wang, Yue Gao, Zhi Lin

**Affiliations:** 1 College of Electronic Countermeasure, National University of Defence Technology, Hefei 230037, China; wyeei@126.com (Y.W.); niuhaonupt@foxmail.com (H.N.); pine_zhao@yeah.net (Q.Z.); wanghfe@163.com (L.W.); 2 Anhui Province Key Laboratory of Electronic Restriction, Hefei 230037, China; 3 School of Computer Science, Fudan University, Shanghai 200433, China; gao_yue@fudan.edu.cn

**Keywords:** NC-OFDM, satellite communications, compressed spectrum, hybrid genetic algorithm particle swarm optimization

## Abstract

With the fast development of giant LEO constellations, the effective spectrum utilization has been regarded as one of the key orientations for satellite communications. This paper focuses on improving the spectrum utilization efficiency of satellite communications by proposing a non-continuous orthogonal frequency division multiplexing (NC-OFDM) method. Based on the models of NC-OFDM system, we first propose a sub-carrier allocation method by using spectrum sensing to efficiently perceive and utilize the spectrum holes in the satellite communication system. Then, a hybrid genetic particle swarm optimization method is adopted to allocate the channel resources effectively. Finally, simulation results verify that the proposed algorithm can significantly improve the spectrum efficiency of satellite communications.

## 1. Introduction

Recently, cognitive radio technology has been regarded as a promising solution for solving spectrum resource shortages due to its characteristic of efficient spectrum utilization [1,2,3]. In addition, satellite communications have received significant attention due to their widely applicability in emergency rescues, environmental monitoring, and network access in unpopulated areas [4,5,6,7,8,9,10,11,12]. The spectrum management of most commercial geostationary orbit communication satellites follows fixed channel authorization allocation modes. With the increase in message volume and the number of users, the frequency band is becoming more and more crowded, and the spectrum resources are increasingly scarce. However, satellite communication channels are not occupied by users all the time, resulting in a large number of spectrum holes in the spectrum of satellite communication. In a report written by the Spectrum Policy Task Force [13], the FCC noted that the spectrum utilization in the vast majority of the authorized spectrum allocated is only 15 to 85%. The report points out the prominent contradictions in spectrum management: spectrum resources are scarce, but the spectrum utilization rate is very low.

NC-OFDM uses cognitive radio technology to discover the spectrum holes in the communication environment and to obtain access to them in the form of non-continuous subcarriers, which avoids the interference from cognitive users to authorized users [14,15]. Other multiple access methods, such as spread spectrum, cannot use spectrum holes due to their continuous spectrum characteristics. The NC-OFDM-related research mainly focuses on spectrum forming [16], channel estimation [17], synchronous [18], peak ratio suppression [19], etc., but there is no detailed solution for NC-OFDM satellite communication proposed. OFDM technology is proposed to be applied to the field of satellite communication, and OFDM communication satellites will be developed [20,21]. Recently, in [22], the authors proposed using NC-OFDM in satellite communication to improve the spectrum efficiency. However, the cost of launching OFDM communication satellites is high. An NC-OFDM system based on cognitive radio can achieve spectrum sharing with authorized users, so it can greatly improve the spectrum utilization. If NC-OFDM can be combined with satellite communication, the spectrum utilization efficiency of satellite communication will be greatly improved, and the shortage of spectrum resources will be alleviated.

The most valuable resource in wireless communication is frequency spectrum, and the rational utilization, sharing, and protection of the radio spectrum has become a growing requirement. It is obviously an economical and feasible means to apply NC-OFDM to satellite communication, which will improve the spectrum utilization efficiency of satellite communication.

## 2. NC-OFDM Satellite Communication System Model Based on Spectrum Sensing

**Figure 1:**
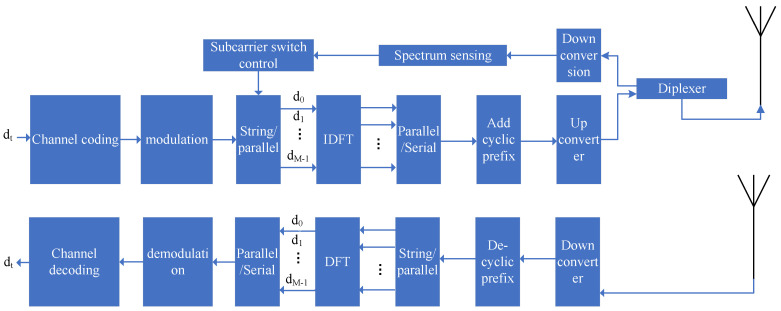
NC-OFDM satellite communication system model based on spectrum sensing.

The NC-OFDM satellite communication system based on cognitive radio is a new type of communication system. In addition to the non-continuous subcarrier functions and the up-and-downlink data transmission functions of satellite communication systems, a spectrum-sensing module is specifically designed. This module can panoramically perceive the spectrum usage of communication satellites and detect spectrum holes. It reasonably allocates non-continuous subcarriers according to the number and frequency band range of the spectrum holes, and sends the dynamic subcarrier allocation scheme to the subcarrier control module in real time for spectrum forming. The basic model of the transmission and reception system is shown in Figure 1.

As shown in Figure 1, the transmit source undergoes channel coding and PSK/QAM modulation, and then it is converted to a set of parallel data $d_{0},d_{1},\ldots ,d_{M-1}$, where *M* represents the available number of subcarriers in the satellite channel perceived by the spectrum-sensing module. Just like the implementation principle of the OFDM system, the modulation of NC-OFDM can be equivalently realized by Inverse Discrete Fourier Transform (IDFT). The frequencies of the NC-OFDM subcarriers are not continuously distributed because they are determined by the number of spectrum holes and the subcarrier allocation algorithm.

By denoting the number of all subcarriers to be allocated in the satellite channel as *N*, the NC-OFDM sampling signal can be obtained by *N*-point IFFT:

(1) $$s_{k}=\sum_{i\in \left\{0,1,2,...,N-1\right\}}d_{i}\exp\left(j\frac{2\pi ik}{N}\right).$$

Similarly, at the receiving terminal, $s_{k}$ can be inversely transformed to restore the original data $d_{i}$:

(2) $$d_{i}=\sum_{k=0}^{N-1}s_{k}\exp\left(-j\frac{2\pi ik}{N}\right).$$

## 3. Compressed Spectrum Sensing

Currently, the frequency range used by one Ku-band communication satellite is about 500 MHz. According to the monitoring results of actual satellite communication frequency occupation, frequency bandwidths of spectrum holes are in the range of about 10 MHz to 100 MHz. Considering the system control complexity brought by the increasing number of subcarriers and the full utilization of spectrum holes, we set the bandwidth of each NC-OFDM subcarrier to 4.125 MHz. So, 500 MHz of bandwidth can be allocated to 122 subcarriers, which include 100 data subcarriers, 12 pilot subcarriers, and 10 protection subcarriers, as shown in Table 1.

Traditional spectrum sensing methods used for cognitive radio include classic energy detection methods [16], cyclic spectrum estimation [23], etc., but these methods are manly used for narrowband communication. On the task of simultaneously monitoring all the subchannels among the 500 MHz bandwidth, several challenges arise such as power consumption and cost of high-speed analog-to-digital converters (ADCs). In recent years, compressed sensing has been proposed for broadband spectrum signal monitoring and multiband signal acquisition [24,25,26]. With a periodic non-uniform sampling framework working far below the Nyquist rate, the signal processing platform based on a software-defined radio can realize real-time, full-band spectrum monitoring within 2 GHz bandwidth, and the spectrum monitoring results can be directly used for spectrum holes extraction and subcarriers allocation.

We hereby propose to integrate the sub-Nyquist sampling and spectrum sensing scheme into the NC-OFDM satellite communication. We adopt a period non-uniform sampling (PNS, also known as Multicoset sampling) architecture. In the PNS, the baseband signal x(t) is sampled by an interleaved ADC array composed of *p* low-speed ADCs working under the sampling rate of $f_{Nyq/L}$, where $f_{Nyq}$ is the Nyquist frequency of the signal and p<L. By setting the analog delay in the *i*-th way as cicifNyqfNyq, where $c_{i}$ is a non-negative integer smaller than *L*, the sampling sequence in the *i*-th way can be expressed by Equation:

(3) $$x_{i}\left[n\right]=x\left(\frac{nL}{f_{Nyq}}+\frac{c_{i}}{f_{Nyq}}\right),\;n\;=\;1,2,\ldots .$$

The *i*-th way of PNS gives the following measurement:

(4) $$Y_{i}\left(f\right)=\frac{L}{f_{Nyq}}\;exp\left(-j2\pi \frac{c_{i}}{L}f\right)X_{i}\left(\;exp\left(j2\pi \;f\frac{L}{f_{Nyq}}\right)\right),$$

where $X_{i}\left(\exp\left(j2\pi \;f\frac{L}{f_{Nyq}}\right)\right)$ is the discrete time Fourier transform (DTFT) of $x_{i}\left[n\right]$. A linear relationship between the measurement and the original spectrum $X\left(f\right)$ is given by Equation (5):

(5) $$y\left(f\right)={\left[Y_{1}\left(f\right)Y_{2}\left(f\right)\ldots Y_{p}\left(f\right)\right]}^{T},$$

where:

(6) $$x\left(f\right)={\left[X\left(f+\frac{\left(-L/2\right)f_{Nyq}}{L}\right)X\left(f+\frac{\left(-L/2+1\right)f_{Nyq}}{L}\right)\ldots \left(f+\frac{\left(L/2-1\right)f_{Nyq}}{L}\right)\right]}^{T}$$

and A is a p×L matrix with its $\left(i,j\right)$-th element being $a_{i,l}=e^{j2\pi \frac{c_{i}}{L}\left(-2/L+l-1\right)}$.

The above equation is underdetermined and has infinite solutions. However, by taking advantage of the sparse nature of the spectrum, we can transform the problem into the following $l_{0}$-norm minimization problem:

(7) $$\underset{\hat{\theta }\left(f\right)\in C}{\arg\min\;}{\left|\hat{\theta }\left(f\right)\right|}_{0}\;\;s.t.\;\;{\left|y\left(f\right)-A\hat{\theta }\left(f\right)\right|}_{2}<\epsilon .$$

This optimization problem is NP-hard but can be approximated by several methods within polynomial time, e.g., convex optimization methods such as basis pursuit (BP), greedy algorithms such as orthogonal matching pursuit (OMP), as well as sparse Bayesian learning algorithm. Among the approaches, greedy algorithms cost far less computation burden than optimization algorithms and yield acceptable solution in high-SNR scenarios. Thus, greedy algorithms, instead of optimization algorithms, are usually adopted in the practical system for better real-time performance. With the above sub-Nyquist sampling and spectrum reconstruction scheme, the average sampling rate acquired by the Ku-band satellites can be significantly reduced.

**Figure 2:**
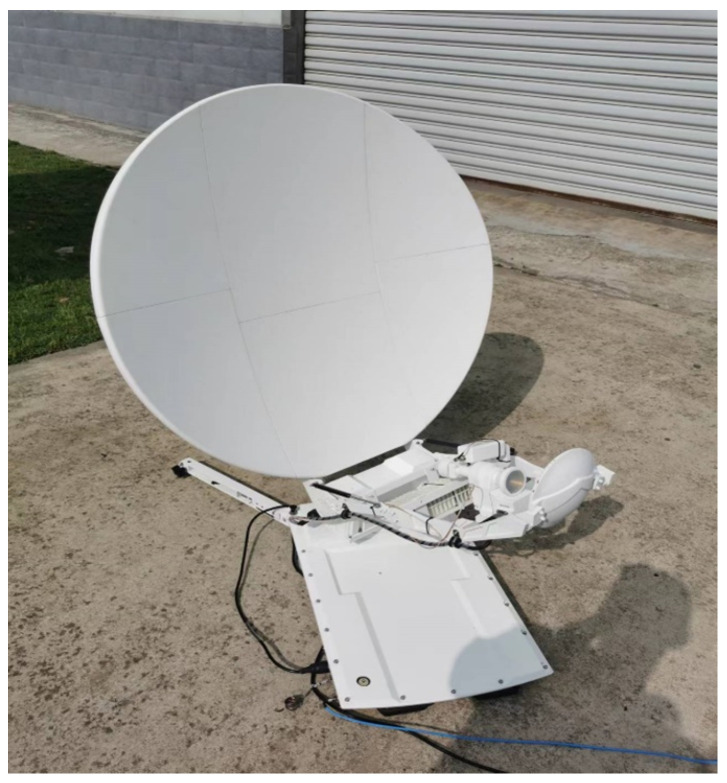
The satellite ground station.

**Figure 3:**
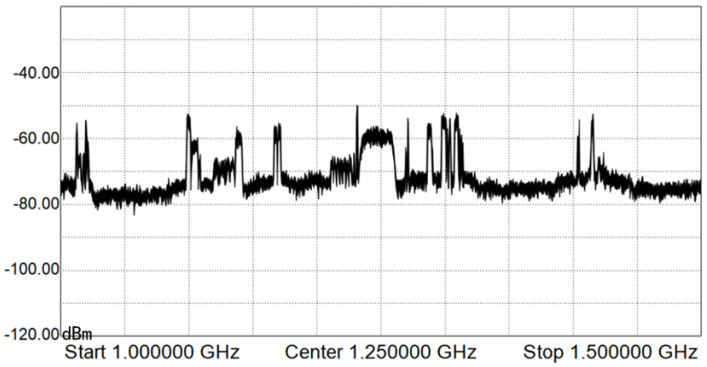
Spectrum sensing results of Ku-band satellite.

We used a satellite ground station to monitor a Ku satellite, as shown in Figure 2, and obtained the real star signal spectrum of the satellite in the 500 MHz band range(from 12,300 MHz to 12,800 MHz). The frequency became 1–1.5 GHz after antenna reception and down-frequency conversion amplification (as shown in Figure 3). Six unused spectrum holes were extracted which occupied spectrum band of 296 MHz. Moreover, 69 subcarriers can be allocated in these unused spectra (as shown in Table 2).

## 4. Subcarrier Power Allocation Algorithm

NC-OFDM is an unlicensed access system based on cognitive radio. The system needs reasonable resource allocation to maximize the spectrum utilization. Because the bottom noise temperature of each satellite spectrum hole monitored by the spectrum and the sub-carrier channel gain varies, the resource allocation strategy, particularly the power allocation strategy, is a key technology for NC-OFDM cognitive users to achieve high-speed communication.

### 4.1. The Description of Power Allocation Problem

In these 69 subcarriers, as shown in Table 2, 57 data subcarriers, 6 pilot subcarriers, and 6 protective subcarriers are allocated. According to Figure 3, the noise temperature of each spectrum hole is different, and the difference between the highest and the lowest value is about 6 dB. The demodulation threshold of BPSK modulation plus convolution code used in satellite communication is about 5 to 7 dB. Thus, we set the SNR of NC-OFDM subcarriers as 7–13 dB, as shown in Table 3.

The problem solved by the power allocation algorithm is how to allocate and optimize the subcarrier power to achieve maximum system throughput within limited total power, known occupancy of authorized user frequency bands, and noise temperature constraints. The authors of [27] proposed a distributed power allocation scheme, which is suitable for resource allocation under multi-user and multi-channel conditions, but little consideration is given to the interference problem of authorized users. In [28], a distributed resource allocation scheme was proposed, which proved that the Particle Swarm Optimization (PSO) algorithm and the dual Lagrangian optimization method have similar performance and acceptable computational complexity. The authors of [29] proposed a method of combining the PSO algorithm and the case-based reasoning method for power allocation, which can speed up the convergence, but the case library samples require time and cost accumulation. In view of the high complexity of the current NC-OFDM sub-carrier power allocation algorithms, a sub-optimal power allocation method based on Hybrid Genetic Particle Swarm Optimization (HGAPSO) algorithm is proposed in this paper.

The NC-OFDM sub-carrier power allocation scheme is converted into an optimization problem, and the maximum overall throughput of cognitive users in the system is taken as the goal of the optimization. The input optimization algorithm of the power allocation scheme was derived, and the scheme was optimized according to the output analysis of the target. The total number of cognitive users is taken as the optimized objective function according to the transmission rate, which is expressed as Formula (8):

(8) $$\max_{P_{i}}\sum_{i=1}^{N}\log_{2}\left(1+\frac{{\left|h_{i}^{SS}\right|}^{2}P_{i}}{\sigma^{2}+J_{i}}\right).$$

The constraint conditions of the objective function are shown in Equation (9):

(9) $$\sum_{i=1}^{N}P_{i}\leq P_{\max},\sum_{i=1}^{N}I_{i}\left(P_{i}\right)\leq I_{th},$$

where $P_{i}$ is the power of the *i*-th sub-carrier, $h_{i}^{SS}$ is the channel gain coefficient between the subcarriers, and $\sigma_{2}$ is the variance in Gaussian noise. $J_{i}$ is the interference noise power of the authorized user to the *i*-th subcarrier:

(10) JiPU=∫fi−ΔfΔf22fi+ΔfΔf2212πN∫−ππΦPU(f)sin(N2(2πf−Φ))sin(12(2πf−Φ))dΦ,

where $\Phi_{PU}\left(f\right)$ is the power spectral density function of the authorized user, $f_{i}$ is the center frequency of the authorized user, and Δf is the bandwidth of the cognitive user. The interference power brought by cognitive users to authorized users is shown in Equation (11):

(11) Ii(Pi)=PihSP2Ts∫fi−BPBP22fi+BPBP22sin(πfTS)πfTS2df,

where $B_{P}$ is the bandwidth of the authorized user, and $h^{SP}$ is the channel gain coefficient introduced by the cognitive user to the authorized user.

### 4.2. Hybrid Genetic Particle Swarm Optimization Algorithm for Power Distribution

Power allocation can be solved by using an optimization algorithm, and the design of the algorithm is the key to the overall performance of cognitive users. At present, optimization algorithms are widely studied, including Genetic Algorithms (GA), PSOs, simulated annealing algorithms, tabu search algorithms, differential evolutionary algorithms, etc., among which GAs and PSOs are the two most widely used algorithms. GAs are a kind of random optimization search method based on the evolutionary rules of the biological world. They can use the search information of multiple search points at the same time. They has the characteristics of parallel operation and are suitable for large-scale complex optimization problems. The factor that has an impact on fitness is the subcarrier power. Therefore, the subcarrier power sequence is adopted as the chromosome in a GA. When the fitness function meets the output requirements, the algorithm outputs the sub-optimization results through initialization, coding, selection, crossover, compilation, and other basic operations.

The idea of the GA comes from natural selection theory, and it has certain advantages for obtaining the optimal solution by searching for the most eligible individuals in the global space based on the rules of survival of the fittest. From the perspective of evolutionary iterations, the PSO algorithm makes use of the information interaction and individual memory within the population to speed up the search. In this paper, combined with the advantages of the two algorithms, a hybrid genetic particle swarm optimization algorithm is proposed. The evolutionary idea of the PSO algorithm is integrated into the updated GA, and the algorithm optimization is achieved by using the competition between the two algorithms.

**Figure 4:**
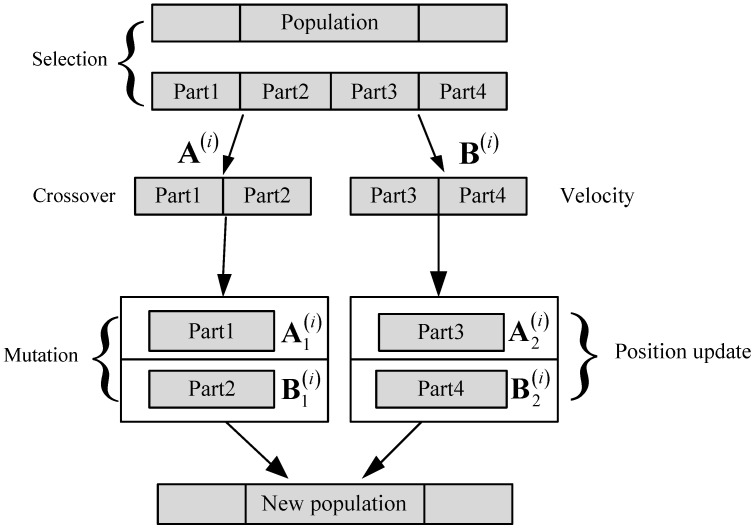
The flow of the HGAPSO.

**Figure 5:**
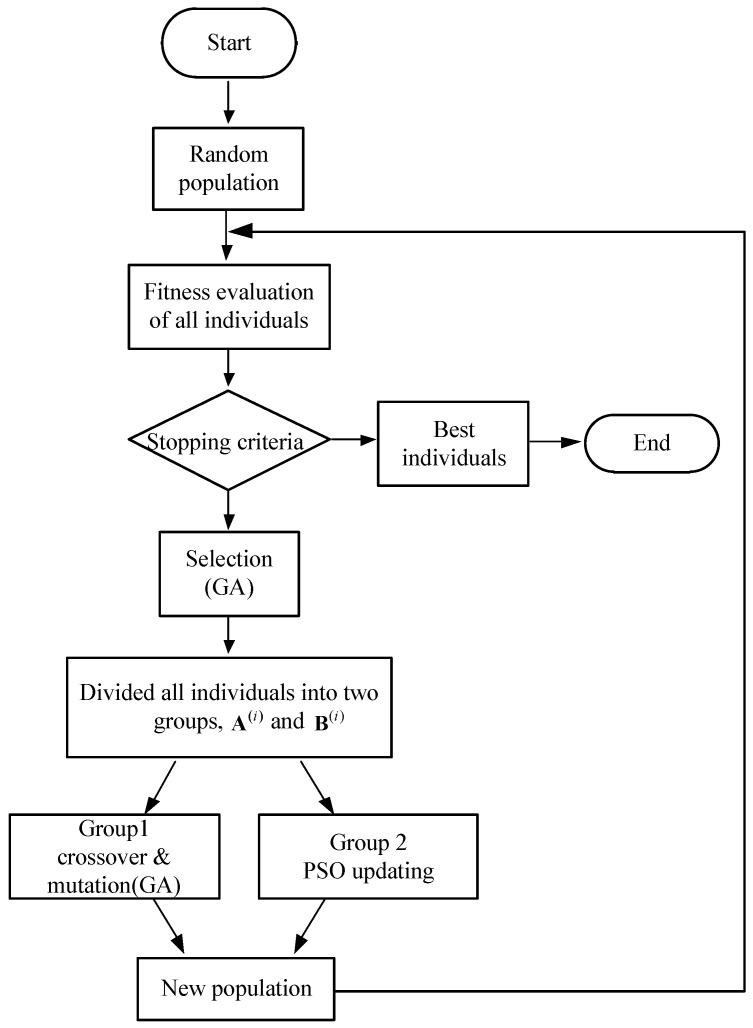
Optimization process of the HGAPSO algorithm.

The flow and update process of the HGAPSO algorithm are shown in Figure 4 and Figure 5, respectively.

The specific optimization steps of HGAPSO are shown in Algorithm 1.
**Algorithm 1:** HGAPSO.1:Set particle update speed, population size, number of particles, weight factor $c_{1}$, weight factor $c_{2}$, coding method, crossover probability, mutation probability;2:Generate a population randomly, initialize particle position, particle speed, and calculate its fitness;3:In the population, find the global best and the best individual and calculate the corresponding best fitness value; randomly divide the population into two categories, defined as $A^{\left(i\right)}$ and $B^{\left(i\right)}$;4:For each group, it is divided into two groups according to the degree of fitness, one of which has a fitness greater than two. Among them, form a new population using GA for crossover and mutation operations; $A_{2}^{\left(i\right)}$ and $B_{2}^{\left(i\right)}$ form another population, using PSO algorithm to update particle speed and position;5:Form a new population after completing the update together, and recalculate the fitness value;6:Judge whether the optimal population meets the requirements or not, then return to step 4, and output the result if it is satisfied.

### 4.3. Analysis of Algorithm Performance

In order to analyze the performance of optimal algorithms for power allocation, 2000 Monte Carlo simulations were conducted for each algorithm. Assuming that the simulation scenario contains 10 cognitive users and 1 authorized user, the signal-to-noise ratio (SNR) of the channel is 0 dB. In order to ensure that each iteration operation time of the three algorithms is similar, the parameters adopted in the simulation are shown in Table 4.

**Figure 6:**
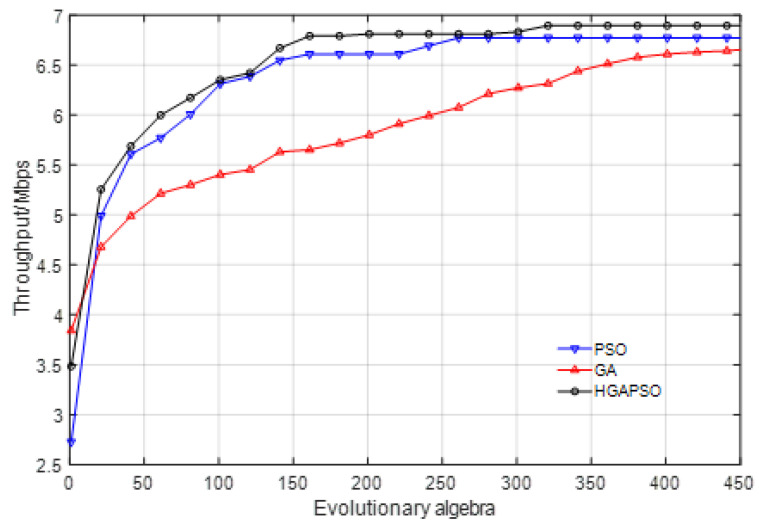
Comparison of the throughput by different algorithms.

Figure 6 shows the system throughput changes generated by iterative operation simulation using three algorithms. As can be seen from the comparison of curves in Figure 6, the HGAPSO algorithm achieves the highest system throughput under the same number of evolution, followed by GA, and the PSO algorithm has the worst performance. After hundreds of iterations, the three algorithms converge on their best performances, among which, the peak throughput of the HGAPSO algorithm is 5.325 Mbps, and that of the GA and PSO are 5.077 and 4.496, respectively. This result shows that under the same evolutionary algebra and complexity, the HGAPSO power allocation algorithm achieves the maximum cognitive user throughput, which is improved by 4.88% and 18.44%, respectively, compared with the GA and the PSO algorithm.

## 5. Simulation of NC-OFDM Satellite Communication System

### 5.1. BER Performance of NC-OFDM Satellite Communication System

**Figure 7:**
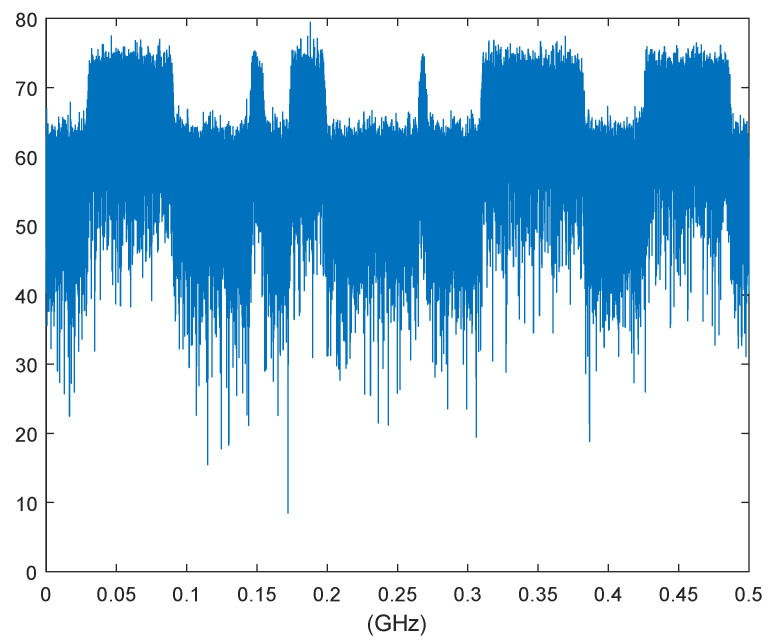
Spectrum of data subcarriers of NC-OFDM.

In order to avoid interference from the subcarrier side band to the adjacent frequencies of authorized users, a frequency domain filter is applied before emission on each subcarrier signal. The spectrum of the IF signal composed of 57 data subcarriers is shown in Figure 7.

**Figure 8:**
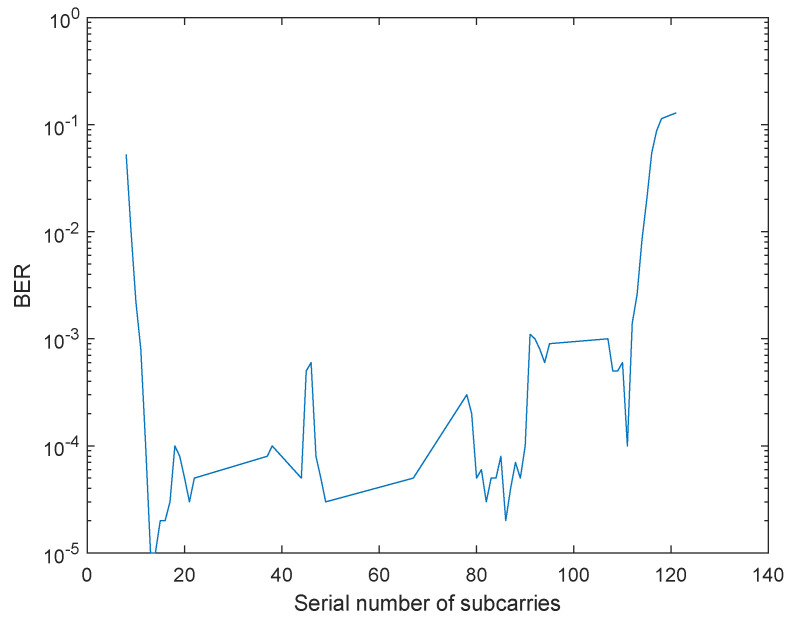
BER of data subcarriers of NC-OFDM.

The channel coding adopts convolutional code 2,1,6, and the modulationtype is BPSK. The transmission and reception of all data subcarriers are simulated and the BER performance is obtained as shown in Figure 8.

The BER threshold of satellite communication is usually $10^{-3}$–$10^{-4}$. As shown in Table 4, 11 subcarriers do not meet the requirements. The reason is that their frequencies are at the up and down boundary of the entire data subcarrier frequency domain, which cannot offset the interference of subcarriers efficiently as OFDM. It can be seen that 57 effective data subcarriers realize a data transmission rate of 228 Mbps, which proves that the NC-OFDM system can make efficient use of the spectrum holes in satellite communication.

### 5.2. Interference Analysis of NC-OFDM Signal to Authorized Signal

The Spectrum Overlap Ratio (SOR) ΔF is defined as the ratio of the spectrum overlapping range between an authorized signal and an NC-OFDM signal to the frequency bandwidth of the former. We suppose that the carrier frequency of the authorized signal is $f_{c1}$, the code rate is $f_{d1}$, the carrier frequency of the adjacent NC-OFDM subcarrier is $f_{c2}$, and the code rate is $f_{d2}$, and they all use MPSK modulation. Therefore:

(12) $$\Delta F=\frac{f_{d1}+f_{d2}-\left|f_{c1}-f_{c2}\right|}{2f_{d1}}$$

**Figure 9:**
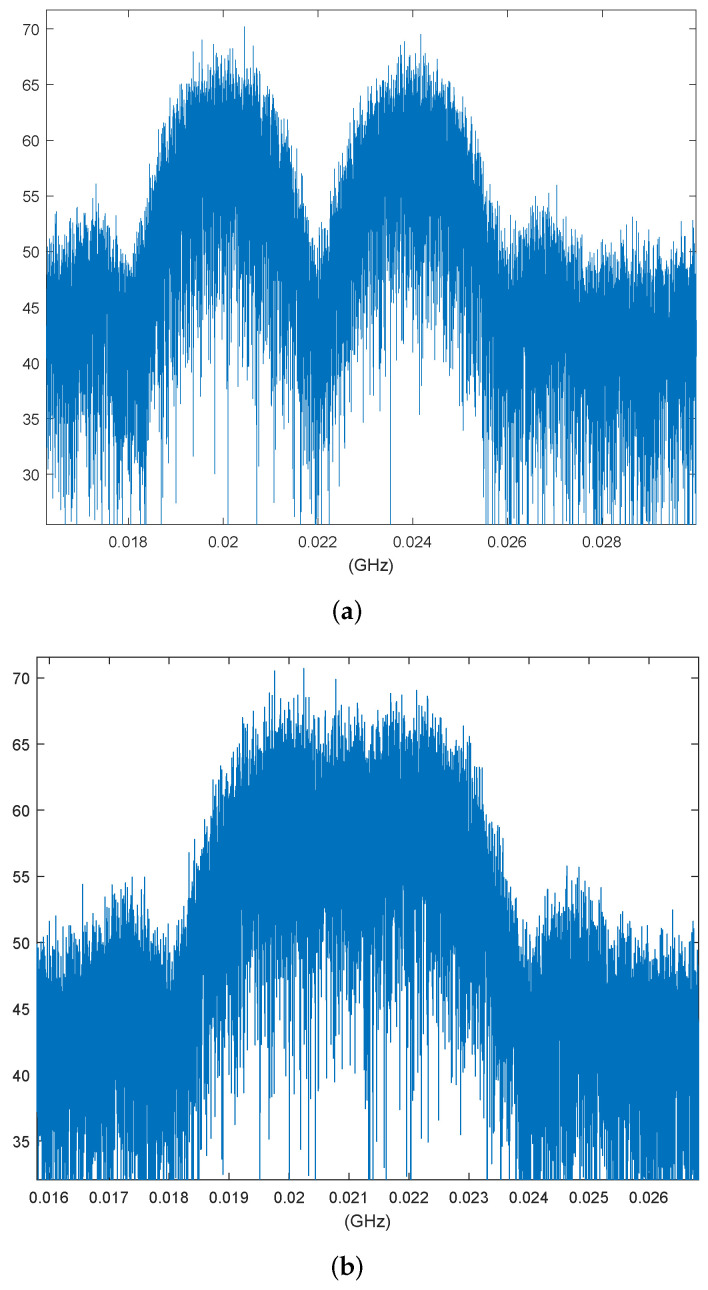
Spectra of QPSK authorized signal and adjacent subcarrier of NC-OFDM: (**a**) Spectrum with ΔF=0; (**b**) Spectrum with ΔF=50%.

We assume that the NC-OFDM signal can make full use of the subchannel gain and the power spectrum densities of subcarriers approach to authorized signal, and both the modulation rate of the authorized signal and the NC-OFDM subcarrier rate are 4MBD. When ΔF equals 0 and 50%, the spectrum of the authorized signal and the adjacent NC-OFDM subcarrier are shown in Figure 9.

**Figure 10:**
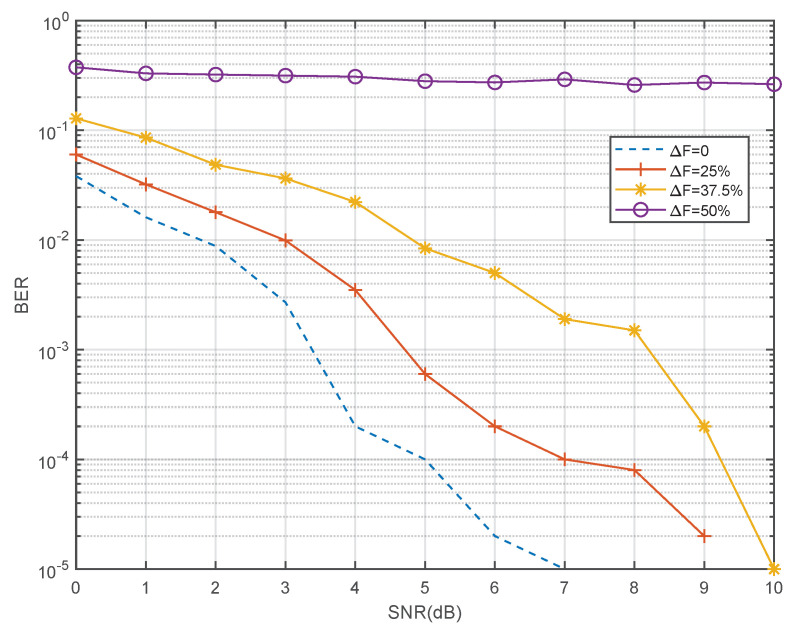
BER of authorized signal with NC-OFDM interference.

Under the conditions of ΔF as 0, 25%, 37.5%, and 50% respectively, the reception BER results of authorized signal with BPSK modulation and convolution coding are shown in Figure 10.

## 6. Conclusions

NC-OFDM communication has advantages of flexible and controllable subcarriers, and it is a promising technique to improve satellite communication spectrum efficiency. The key issue of whether NC-OFDM can be applied is its compatibility with authorized satellite communication users.

Based on the compressed spectrum sensing of a real satellite channel, a subcarrier distribution scheme for NC-OFDM satellite communication is proposed in this paper. Moreover, a hybrid genetic particle swarm optimization algorithm is proposed for NC-OFDM sub-carrier power control, which maximizes the throughput of the NC-OFDM system. Through the effective utilization of the satellite spectrum holes, the extra data transmission of 228 Mbps is achieved, which is impressive for commercial communication satellites with a bandwidth of 500 MHz. The concept of the spectrum overlap ratio is defined to describe the interference of NC-OFDM signals to authorized signals. The BER performance of authorized system under interference condition is studied. Fast and efficient spectrum sensing and flexible power allocation of subcarriers are effective ways to solve the compatibility problem. The NC-OFDM satellite communication method can significantly improve the spectrum efficiency of satellite communication. This work is an extended version of the previously published conference paper [22].

## Figures and Tables

**Table 1:** Subcarriers of Ku satellite with 500 MHz frequency band.

Subcarrier Division Parameters	Definition
number of data subcarriers	100
number of pilot subcarriers	12
number of guard subcarriers	10
total number of subcarriers	122
subcarrier frequency spacing	4.125 MHz

**Table 2:** Spectrum holes extracting and subcarrier allocation.

Spectrum Hole Number	Start Frequency (MHz)	Cut-Off Frequency (MHz)	Bandwidth (MHz)	Assign Sub-Carrier Number
1	12,325	12,395	70	7–23
2	12,445	12,465	20	36–39
3	12,475	12,510	35	43–50
4	12,568	12,582	14	66–68
5	12,615	12,700	85	77–96
6	12,728	12,800	72	106–122

**Table 3:** SNR of data subcarriers.

Subcarrier Number	SNR (dB)	Subcarrier Number	SNR (dB)
8	12	67	7
9–19	13	78–79	7
20	13	80–90	10
21	12	91–95	7
22	11	107	7
37	9	108	8
38	8	109	9
44–46	7	110–119	10
47–49	9	120–121	9

**Table 4:** Parameters of different algorithms.

Algorithm	Parameters
GA	Evolutionary algebra: 200, population size: 300, crossover probability: 0.3, mutation probability: 0.05
PSO	Number of iterations: 200, population size: 60, particle speed: 2, weight factor $c_{1}$: 0.5, weight factor $c_{2}$: 1.5
HGAPSO	Evolutionary algebra: 200, population size: 100, particle velocity: 2, weight factor $c_{1}$: 0.5, weight factor $c_{2}$: 1.5, crossover probability: 0.3, mutation probability: 0.05
